# TELEREHABILITATION VERSUS FACE-TO-FACE PHYSICAL THERAPY FOR MIDDLE-AGED PATIENTS WITH DEGENERATIVE MENISCAL TEAR IN CHINA: A NON-INFERIORITY RANDOMIZED CONTROLLED TRIAL

**DOI:** 10.2340/jrm.v57.43237

**Published:** 2025-08-21

**Authors:** Jiye HE, Caiqi XU, Lihua HUANG, Hui WANG, Shengdi LU

**Affiliations:** 1 Department of Orthopedics, Shanghai Xinhua Hospital, Shanghai; 2 Department of Sports Medicine, Shanghai Sixth People’s Hospital, Shanghai; 3 Department of Rehabilitation, Shanghai Sixth People’s Hospital, Shanghai; 4 Department of Orthopedics, Shanghai Sixth People’s Hospital, Shanghai, China

**Keywords:** cost–benefit analysis, knee injuries, meniscal tear, physical therapy modalities, telerehabilitation, randomized controlled trial

## Abstract

**Objective:**

This study aimed to compare the effectiveness of telerehabilitation to traditional face- to-face physical therapy for patients with degenerative meniscal tears in Shanghai, China.

**Design:**

A two-arm, single-blinded, randomized controlled trial was conducted across two hospitals in Shanghai, China.

**Subjects/Patients:**

Participants with clinically diagnosed degenerative meniscal tears were randomly assigned to either the telerehabilitation group or the face-to-face physical therapy group.

**Methods:**

Both groups underwent a 12-week intervention. Participants in telerehabilitation group used a digital platform for remote physical therapy, while participants in physical therapy group received traditional clinic-based rehabilitation. A predefined non-inferiority margin of 10 points on the Knee Injury and Osteoarthritis Outcome Score (KOOS) was applied to determine clinical equivalence between the interventions.

**Results:**

Both groups demonstrated significant improvements in knee function and quality of life, with no significant differences in the KOOS, SF-36, or functional tests at any time point (*p* > 0.05). Both groups had high adherence rates, with no significant differences in exercise completion or satisfaction scores. The TELE group had a significantly lower total cost compared to the PT group (*p* < 0.001), demonstrating greater cost-effectiveness.

**Conclusion:**

Telerehabilitation was found to be clinically non-inferior to face-to-face physical therapy for improving knee function, pain, and quality of life in patients with degenerative meniscal tears. It offered significant cost savings, making it a cost-effective alternative to traditional in-person rehabilitation.

## LAY ABSTRACT

This study aimed to compare the effectiveness of telerehabilitation with traditional in-person physical therapy for patients in Shanghai, China, suffering from degenerative meniscal tears. In a randomized controlled trial conducted across 2 hospitals, participants were assigned to either the telerehabilitation group or the in-person physical therapy group. Over 12 weeks, both groups received their respective interventions. The results showed that both groups experienced significant improvements in knee function and quality of life, with no notable differences between them. Adherence to the exercise programmes was high in both groups, and satisfaction levels were comparable. However, the telerehabilitation group incurred significantly lower costs, indicating greater cost-effectiveness. In conclusion, telerehabilitation was found to be as effective as traditional in-person physical therapy in improving knee function, pain, and quality of life for patients with degenerative meniscal tears. Additionally, it offered significant cost savings, making it a viable alternative to traditional in-person rehabilitation.

Degenerative meniscal tears are a prevalent condition among middle-aged patients, leading to knee pain, functional limitations, and reduced quality of life (1). Traditional face-to-face physical therapy has been a cornerstone in managing these conditions, demonstrating effectiveness in pain relief and functional recovery. However, the increasing demand for accessible healthcare services has propelled the adoption of telerehabilitation, a digital alternative enabling remote delivery of physical therapy interventions.

Globally, telerehabilitation has been applied successfully in various musculoskeletal conditions. In Italy, treatment algorithms incorporating continuous low-level heat-wrap therapy via telerehabilitation platforms have been developed to manage musculoskeletal pain effectively (2). Such approaches reduce the dependency on in-person visits while maintaining therapeutic efficacy. Similarly, research highlights barriers and facilitators to telerehabilitation adoption, including infrastructure challenges and patient acceptability (3). These findings underline the potential of telerehabilitation to bridge care gaps, especially in resource-constrained or geographically diverse regions.

While telerehabilitation has gained traction worldwide, its application in China remains underexplored. In China, rapid urbanization, significant rural–urban healthcare disparities, and an ageing population create a pressing need for innovative, accessible rehabilitation solutions, yet research on telerehabilitation in Chinese populations remains scarce despite widespread smartphone use and robust digital infrastructure. For some common diseases, like degenerative meniscal tears, limited studies have evaluated their effectiveness in Chinese populations, emphasizing the need for localized research to address cultural and systemic healthcare differences. Despite this, the broader application of telemedicine in managing chronic diseases in China indicates a growing acceptance of digital healthcare solutions (4).

This study aims to compare the effectiveness of telerehabilitation with traditional face-to-face physical therapy in managing degenerative meniscal tears among middle-aged patients in China. The primary objective is to establish non-inferiority in functional outcomes and patient satisfaction between these modalities, contributing evidence to the global discourse on digital healthcare innovations. We hypothesize that telerehabilitation is non-inferior to face-to-face physical therapy in improving pain, function, and quality of life among middle-aged Chinese patients with degenerative meniscal tears.

## METHODS

### Study design

This was a 2-arm, single-blinded (assessor-blinded), randomized, controlled, 2-centred clinical trial conducted at Shanghai Xinhua Hospital and Shanghai Sixth People’s Hospital. The study adhered to the principles of the Consolidated Standards of Reporting Trials (CONSORT) (5) and was approved by the Ethics Review Committees of Shanghai Sixth People’s Hospital. Other participating institutions and hospitals acknowledged the IRB approval. Recruitment began in January 2020 and concluded in October 2023. Middle-aged patients with degenerative meniscal tears were randomized to 1 of 2 groups: telerehabilitation (TELE group) or face-to-face rehabilitation (PT group). Participants received a 12-week intervention and were followed up at 6 weeks, 12 weeks, and 24 weeks. Randomization was performed via a computer-generated sequence with stratification by centre.

### Participants

Inclusion criteria:

- Patients aged 40–65 years with MRI-confirmed degenerative meniscal tears.
- Persistent knee pain for at least 3 months.
- Ability to use a smartphone or smart device.
- Willingness to provide written informed consent.

Exclusion criteria:

- Severe knee osteoarthritis (Kellgren–Lawrence grade ≥ 3).
- Previous knee surgery.
- Coexisting inflammatory arthritis, neurological conditions, or other lower-limb musculoskeletal disorders.

Participants were informed about the study during outpatient visits and screened for eligibility. Informed consent was obtained prior to enrolment.

### Interventions

*Telerehabilitation group (TELE group).* Participants used the Joymotion^TM^ app, a digital platform for remote physical therapy. The app provided daily guided exercise regimens tailored to individual needs, with real-time feedback on performance. Weekly online consultations with a physical therapist were conducted to monitor progress and adjust the exercise programme as needed. Participants completed 12 weeks of intervention at home, with adherence monitored through app usage logs. Participants in the TELE group received the same rehabilitation programme as participants in the PT group (see Appendix S1).

*Face-to-face rehabilitation group (PT group).* Participants attended 3–4 in-clinic sessions per week over 12 weeks. Each session lasted approximately 45–60 min and focused on traditional physical therapy exercises, including strengthening, mobility, and functional training (see Appendix S1). Sessions were supervised by physical therapists, and participants received additional home exercises based on progress assessments.

### Outcomes

*Primary outcome.* The primary outcome was the Knee Injury and Osteoarthritis Outcome Score (KOOS), a validated patient-reported measure designed to assess 5 subdomains: Pain, Symptoms, Activities of Daily Living (ADL), Sport/Recreation, and Quality of Life (QoL). Each item was scored on a 5-point Likert scale, and the total score was transformed to a 0–100 scale, where 100 indicated no symptoms or limitations (6). The primary analysis focused on the overall KOOS score at 12 weeks.

*Secondary outcomes.* Secondary outcomes included various objective and subjective measures. The Physical Component Summary (PCS) and Mental Component Summary (MCS) of the SF-36 were used to assess overall health status, with the PCS reflecting physical health and the MCS reflecting mental health (7). These scores were derived from the 8 domains of the SF-36 and ranged from 0 to 100, where higher scores indicated better health (7). Muscle strength was evaluated through peak torque extension and peak torque flexion, which were measured using an isokinetic dynamometer (8). Participants performed maximal knee extension and flexion movements at angular velocities of 60°/s and 180°/s, and the maximum torque generated by the quadriceps and hamstrings was recorded in Newton-metres (Nm) (8).

Functional performance was assessed using the One-Leg Hop Test and the 6-Meter Timed Hop Test. The One-Leg Hop Test measured the maximum forward hopping distance on one leg in centimetres, reflecting dynamic strength and balance (1). The 6-Meter Timed Hop Test evaluated agility and functional performance by recording the time taken to hop 6 m on one leg in seconds (9). Muscular endurance was evaluated with the Knee Bends in 30-Seconds Test, where participants performed as many full knee bends as possible within 30 s ensuring proper form and full range of motion (10).

A cost-effectiveness analysis compared the economic efficiency of telerehabilitation with face-to-face rehabilitation. This included intervention costs, healthcare utilization, absenteeism, and productivity losses, while effectiveness was measured by the difference in KOOS scores. The incremental cost-effectiveness ratio (ICER) was calculated to determine the cost per unit of health benefit gained. Outcome measures were assessed at baseline, 6 weeks, 12 weeks, and 24 weeks to evaluate progress and compare the effectiveness of the 2 rehabilitation approaches.

Outcome measures were assessed at baseline, 6 weeks, 12 weeks, and 24 weeks.

### Cost-effectiveness analysis (CEA)

Cost-effectiveness was assessed by calculating the incremental cost-effectiveness ratio (ICER) between the TELE group and FTF group. Costs included intervention-related costs, healthcare resource utilization, and productivity losses due to absenteeism and presenteeism. Data were collected from hospital records and patient-reported logs. CEA was conducted based on the outcomes at 12 weeks’ follow-up (at the end of the intervention).

### Sample size calculation

The sample size was calculated to detect a non-inferiority margin of 10 points on the KOOS, assuming a standard deviation of 15 points. A total of 120 participants (60 per group) were required to achieve 80% power at a 1-sided alpha level of 0.025, accounting for a 10% dropout rate.

### Randomization and blinding

Middle-aged patients referred to Shanghai Xinhua Hospital and Shanghai Sixth People’s Hospital suspected of having degenerative meniscus tear were informed about the study. At the second outpatient visit (confirmed by MRI scan), after written informed consent, we randomized eligible patients to either the TELE group or the PT group using a central computer-generated randomization scheme in a 1:1 ratio with random blocks (maximum block size of 6).

The sequence was managed by a research assistant not involved in the study. Outcome assessors were blinded to group allocation to minimize bias. Participants, physicians, and physical therapists were not blinded.

*Statistical analysis* (See Appendix S1)

## RESULTS

### Patients

**Fig. 1:**
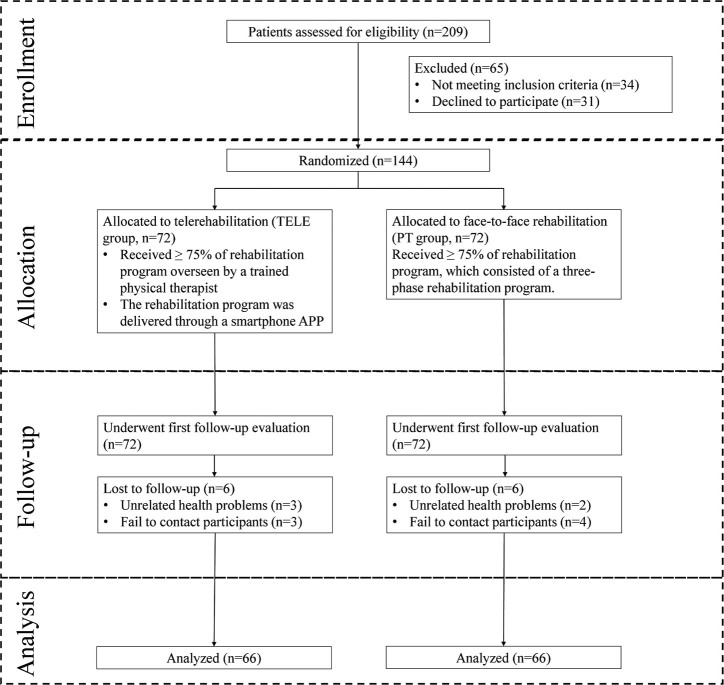
Flowchart of the study, from enrolment to analysis.

We evaluated 209 middle-aged patients with degenerative meniscus injury and 144 were randomized. A flowchart of our study is specified in Fig. 1. The baseline characteristics of the 2 groups (TELE and PT) in the intention-to-treat population were similar, with no significant differences observed in most variables. The TELE group and PT group included 72 participants each, with approximately equal distributions of male patients (50% vs 51.39%, *p* = 0.868). The mean age was slightly higher in the TELE group (49.24 ± 7.46 years) compared with the PT group (47.79 ± 7.22 years), but this difference was not statistically significant (*p* = 0.240). Body mass index (BMI), smoking status, use of analgesics, and education levels were comparable between groups (Table I).

**Table I:** Baseline characteristics of the TELE group and PT group

Characteristic	TELE group (*n* = 72)	PT group (*n* = 72)	*p*- value
Male patients, *n* (%)	36 (50.00)	37 (51.39)	0.868
Right knee, *n* (%)	25 (34.72)	39 (54.17)	0.019
Age, years, mean ± SD	49.24 ±7.46	47.79 ±7.22	0.240
BMI, kg/m^2^, mean ± SD	23.83 ±2.99	23.20 ±2.71	0.187
Smokers, *n* (%)	50 (69.44)	51 (70.83)	0.856
Use analgesics daily, *n* (%)	9 (12.50)	10 (13.89)	0.806
Education at university level, *n* (%)	30 (41.67)	31 (43.06)	0.866
Kellgren–Lawrence classification			0.571
Grade 0	46 (63.89)	52 (72.22)	
Grade 1	22 (30.56)	17 (23.61)	
Grade 2	3 (4.17)	3 (4.17)	
Grade 3	1 (1.39)	0 (0.00)	
Meniscal degeneration			0.905
Grade 1	3 (4.17)	4 (5.56)	
Grade 2	4 (5.56)	3 (4.17)	
Grade 3a	31 (43.06)	34 (47.22)	
Grade 3b	34 (47.22)	31 (43.06)	
Meniscal extrusion, *n* (%)	39 (54.17)	42 (58.33)	0.614
KOOS scores			
KOOS	54.36 (3.40)	53.69 (3.56)	0.247
Pain	63.16 (5.97)	61.88 (6.03)	0.205
Symptoms	70.63 (8.07)	69.59 (7.12)	0.413
Activities of daily living	74.20 (2.71)	74.71 (3.13)	0.297
Function in sport and recreation	43.82 (4.48)	44.31 (5.89)	0.578
Knee related quality of life	39.84 (5.97)	38.98 (7.15)	0.240
SF-36 points			
Physical component summary	47.92 (12.94)	48.06 (14.84)	0.952
Mental component summary	54.97 (9.94)	54.39 (13.50)	0.768
Muscle strength			
Peak torque extension (Nm)	156.09 (16.46)	158.16 (15.68)	0.441
Peak torque flexion (Nm)	81.83 (10.65)	81.61 (10.09)	0.896
Performance tests			
One-leg hop test (cm)	76.61 (4.68)	76.45 (5.24)	0.846
6 m timed hop test (s)	3.15 (0.21)	3.16 (0.21)	0.791
Knee bends 30 s test (*n*)	27.90 (2.46)	27.90 (3.07)	0.994

TELE: home-based telerehabilitation; PT: clinic-based face-to-face rehabilitation; BMI: body mass index; KOOS: Knee Injury and Osteoarthritis Outcome Score; SF-36: Short Form 36 Health Survey.

Knee characteristics, such as the Kellgren–Lawrence classification and meniscal degeneration grades, showed similar distributions across the groups. However, a significant difference was observed in the proportion of patients with right knee involvement (34.72% in TELE vs 54.17% in PT, *p* = 0.019). Baseline KOOS scores, including overall and subdomain scores (pain, symptoms, activities of daily living, sport and recreation function, and knee-related quality of life), did not differ significantly between the groups. Similarly, SF-36 physical and mental component scores, muscle strength measures (peak torque extension and flexion), and performance test results (e.g., one-leg hop test, 6-m timed hop test, and knee bends test) were balanced between the TELE group and PT group, with no significant differences noted (see Table I).

### Primary and secondary outcomes

At 12 weeks post-intervention, improvements in the primary outcome, the Knee Injury and Osteoarthritis Outcome Score (KOOS), were observed in both the TELE group and PT group, with no significant differences between the groups across all time points. At 12 weeks, the adjusted coefficient for KOOS between the TELE group and PT group was 0.483 (95% CI: –0.931 to 1.897, *p* = 0.501). Subdomains of KOOS, including pain, symptoms, activities of daily living, function in sport and recreation, and knee-related quality of life, also improved comparably in both groups. For example, the coefficient for pain at 12 weeks was 1.108 (95% CI: –1.138 to 3.355, *p* = 0.331), while for activities of daily living, it was –0.706 (95% CI: –2.250 to 0.839, *p* = 0.370).

Secondary outcomes, such as the SF-36 Physical and Mental Component Summaries, showed comparable improvements between the TELE group and PT group at all follow-up points. At 12 weeks, the coefficient for the physical component summary was –0.202 (95% CI: –5.110 to 4.706, *p* = 0.935), and for the mental component summary, it was 1.242 (95% CI: –2.958 to 5.443, *p* = 0.560). At 24 weeks, a trend towards greater improvement in the mental component summary was noted in the TELE group, with a coefficient of 1.848 (95% CI: –2.619 to 6.316, *p* = 0.415), but this difference was not statistically significant (Tables II, III).

**Table II:** Changes in outcomes for the TELE and FT groups at weeks 12 after the surgery (in per-protocol population).

Outcome	6 weeks post-surgery			12 weeks post-surgery			24 weeks post-surgery		
	TELE group (*n* = 66)	PT group (*n* = 66)	*p*-value	TELE group (*n* = 66)	PT group (*n* = 66)	*p*-value	TELE group (*n* = 66)	PT group (*n* = 66)	*p*-value
KOOS scores									
KOOS	7.72 (2.52)	7.93 (2.42)	0.642	16.61 (3.76)	16.84 (3.22)	0.710	18.73 (9.97)	17.71 (13.15)	0.620
Pain	5.85 (3.18)	6.44 (2.83)	0.266	11.74 (4.66)	12.37 (4.41)	0.429	11.83 (10.60)	11.03 (13.88)	0.713
Symptoms	7.09 (3.65)	6.11 (3.50)	0.123	12.56 (5.14)	11.26 (4.98)	0.145	11.31 (10.97)	8.93 (15.37)	0.311
Activities of daily living	7.40 (1.58)	7.44 (1.62)	0.874	14.64 (1.94)	14.77 (2.22)	0.716	14.33 (11.08)	13.39 (15.46)	0.692
Function in sport and recreation	7.35 (5.09)	8.26 (5.47)	0.329	16.44 (7.37)	17.58 (7.45)	0.384	21.14 (11.70)	20.91 (14.19)	0.921
Knee-related quality of life	10.61 (6.62)	10.89 (7.08)	0.813	25.66 (10.61)	27.80 (7.74)	0.412	29.73 (15.54)	29.64 (16.37)	0.973
SF-36 points									
Physical component summary	9.17 (4.81)	8.03 (5.50)	0.212	13.56 (7.87)	12.80 (7.74)	0.581	17.12 (13.51)	16.36 (12.20)	0.738
Mental component summary	9.03 (4.14)	8.12 (4.40)	0.227	13.64 (6.60)	11.45 (6.49)	0.060	17.03 (11.19)	13.58 (11.83)	0.090
Muscle strength									
Peak torque extension (Nm)	3.08 (2.00)	4.08 (2.39)	0.011	9.26 (4.50)	11.43 (5.53)	0.016	9.94 (20.00)	9.60 (31.41)	0.942
Peak torque flexion (Nm)	3.78 (2.22)	3.82 (2.36)	0.935	11.39 (5.89)	11.45 (5.92)	0.960	13.86 (14.00)	12.05 (17.50)	0.515
Performance tests									
One-leg hop test (cm)	2.25 (0.93)	2.32 (1.01)	0.667	11.82 (4.88)	11.51 (4.07)	0.697	14.32 (12.49)	11.31 (16.61)	0.245
6 m timed hop test (s)	–0.02 (0.01)	–0.02 (0.01)	0.222	–0.06 (0.02)	–0.07 (0.02)	0.568	–0.13 (0.38)	–0.17 (0.50)	0.562
Knee bends 30 sec test (*n*)	1.13 (0.45)	1.08 (0.47)	0.564	1.73 (0.49)	1.70 (0.61)	0.754	1.80 (4.12)	1.37 (5.20)	0.604

TELE: home-based telerehabilitation; PT: clinic-based face-to-face rehabilitation; KOOS: Knee Injury and Osteoarthritis Outcome Score; SF-36: Short Form 36 Health Survey.

**Table III:** Effectiveness estimates from linear mixed effects models (in per-protocol population).

Outcome	6 weeks post-surgery			12 weeks post-surgery			24 weeks post-surgery		
	Coefficient	95% CI	*p*-value	Coefficient	95% CI	*p*-value	Coefficient	95% CI	*p*-value
KOOS scores									
KOOS	0.525	(–0.761, 1.812)	0.421	0.483	(–0.931, 1.897)	0.501	0.774	(–1.618, 3.165)	0.526
Pain	1.220	(–0.936, 3.376)	0.265	1.108	(–1.138, 3.355)	0.331	1.410	(–1.086, 3.908)	0.266
Symptoms	1.461	(–1.307, 4.230)	0.298	1.732	(–1.070, 4.535)	0.224	2.138	(–0.883, 5.159)	0.164
Activities of daily living	–0.668	(–1.649, 0.312)	0.180	–0.706	(–2.250, 0.839)	0.370	–0.457	(–3.034, 2.120)	0.695
Function in sport and recreation	–0.909	(–2.913, 1.095)	0.371	–1.136	(–3.371, 1.098)	0.316	–0.909	(–3.537, 1.719)	0.495
Knee-related quality of life	0.331	(–2.284, 2.947)	0.802	–0.095	(–2.854, 2.665)	0.946	0.071	(–3.990, 4.132)	0.972
SF-36 points									
Physical component summary	–0.265	(–5.063, 4.533)	0.913	–0.202	(–5.110, 4.706)	0.935	–0.170	(–5.145, 4.805)	0.946
Mental component summary	0.667	(–3.401, 4.734)	0.746	1.242	(–2.958, 5.443)	0.560	1.848	(–2.619, 6.316)	0.415
Muscle strength									
Peak torque extension (Nm)	–4.611	(–10.245, 1.022)	0.108	–5.165	(–10.937, 0.607)	0.079	–4.818	(–11.110, 1.474)	0.132
Peak torque flexion (Nm)	0.312	(–3.423, 4.048)	0.869	0.300	(–3.566, 4.166)	0.878	0.760	(–3.488, 5.008)	0.724
Performance tests									
One-leg hop test (cm)	0.120	(–1.615, 1.856)	0.891	0.235	(–1.600, 2.070)	0.800	0.969	(–1.263, 3.201)	0.392
6 m timed hop test (s)	–0.008	(–0.080, 0.065)	0.836	–0.007	(–0.079, 0.065)	0.855	0.005	(–0.079, 0.089)	0.914
Knee bends 30 sec test (*n*)	–0.109	(–1.049, 0.831)	0.819	–0.107	(–1.062, 0.848)	0.825	–0.006	(–1.034, 1.022)	0.990

TELE: home-based telerehabilitation; PT: clinic-based face-to-face rehabilitation; KOOS: Knee Injury and Osteoarthritis Outcome Score; SF-36: Short Form 36 Health Survey.

Muscle strength outcomes, measured by peak torque extension and flexion, demonstrated significant improvements within both groups. However, the PT group showed greater improvement in peak torque extension at 6 and 12 weeks, with coefficients of –4.611 (95% CI: –10.245 to 1.022, *p* = 0.108) and –5.165 (95% CI: –10.937 to 0.607, *p* = 0.079), respectively. This difference was no longer significant at 24 weeks, with a coefficient of –4.818 (95% CI: –11.110 to 1.474, *p* = 0.132). Peak torque flexion showed no significant differences between groups at any time point, with a coefficient of 0.300 (95% CI: –3.566 to 4.166, *p* = 0.878) at 12 weeks (see Tables II, III).

Performance test outcomes, including the one-leg hop test, 6-m timed hop test, and knee bends in 30 s, improved similarly in both groups without significant differences. At 12 weeks, the coefficient for the one-leg hop test was 0.235 (95% CI: –1.600 to 2.070, *p* = 0.800), and for the 6-m timed hop test, it was –0.007 (95% CI: –0.079 to 0.065, *p* = 0.855). Similarly, for the knee bends in 30 s test, the coefficient at 12 weeks was –0.107 (95% CI: –1.062 to 0.848, *p* = 0.825) (see Tables II, III).

*Cost-effectiveness analysis* (See Appendix S1 and Tables IV, V)

**Table IV:** Average total cost per patient in the TELE and FT groups during the 12 weeks after the surgery

Cost category (CNY)	TELE group (*n* = 66)	PT group (*n* = 66)	*p*- value
Intervention cost			
Rehabilitation cost	6,980.00 (0.00)	10,780.18 (1,475.13)	0.000
Hospital stay cost	7,021.20 (860.90)	6,917.85 (1,066.83)	0.541
Other medical cost			
Primary care	124.24 (46.61)	1,918.18 (262.48)	0.000
Secondary care	5,103.37 (962.70)	6,837.18 (2,156.26)	0.000
Paid home care	591.91 (211.20)	818.48 (268.16)	0.000
Medication	7,207.50 (1,514.13)	7,883.35 (1,348.26)	0.008
Non-medical cost			
Transportation cost	473.11 (188.32)	1,543.64 (282.29)	0.000
Nutrition cost	2,999.71 (504.00)	2,886.95 (482.90)	0.192
Opportunity cost			
Lost wages for patients	23,330.05 (10,668.30)	26,718.38 (1,4629.47)	0.131
Lost wages for families	2,831.26 (3,341.20)	2,312.76 (3,093.54)	0.357
Total cost	5,6662.34 (10,908.35)	68,616.95 (15,072.76)	0.000

TELE: home-based telerehabilitation; PT: clinic-based face-to-face rehabilitation; CNY: Chinese Yuan.

**Table V:** Incremental cost-effectiveness ratio (ICER)

Factor	Main analysis: mixed effects
Incremental cost, CNY	–11954.61
Incremental KOOS score	0.483 (–0.931, 1.897)
Incremental Pain score	1.108 (–1.138, 3.355)
Incremental Symptoms score	1.732 (–1.070, 4.535)
Incremental Activities of daily living score	–0.706 (–2.250, 0.839)
Incremental Function in sport and recreation score	–1.136 (–3.371, 1.098)
Incremental Knee-related quality of life score	–0.095 (–2.854, 2.665)
Incremental Physical component summary score	–0.202 (–5.110, 4.706)
Incremental Mental component summary score	1.242 (–2.958, 5.443)
ICER (KOOS)	–24750.74534
ICER (Pain)	–10789.35921
ICER (Symptoms)	–6902.20
ICER (Activities of daily living)	16932.88
ICER (Function in sport and recreation)	10523.42
ICER (Knee-related quality of life)	125838.00
ICER (Physical component summary)	59181.24
ICER (Mental component summary)	–9625.29

TELE: home-based telerehabilitation; PT: clinic-based face-to-face rehabilitation; KOOS: Knee Injury and Osteoarthritis Outcome Score; KOOS: Knee Injury and Osteoarthritis Outcome Score; ICER: incremental cost-effectiveness ratio.

*Patients’ adherence* (See Appendix S1 and Table SI)

*Adverse events* (See Appendix S1 and Table SII)

## DISCUSSION

This study aimed to evaluate the non-inferiority of telerehabilitation compared with traditional face- to-face physical therapy for patients with degenerative meniscal tears in China. The results showed that both interventions led to significant improvements in knee function, pain, and quality of life, with no significant differences between the 2 groups across primary and secondary outcomes, including the KOOS, SF-36, muscle strength, and performance tests. Furthermore, patient adherence to treatment was comparable, and both groups reported high levels of satisfaction with the intervention. Importantly, the TELE group demonstrated significant cost savings, with lower rehabilitation, primary care, transportation, and other medical costs compared with the PT group. These findings suggest that telerehabilitation is not only clinically effective but also a more cost-effective alternative to traditional in-person rehabilitation, providing an important option for healthcare systems with resource limitations.

Our results indicated that both the TELE group and PT group experienced significant improvements in knee function and quality of life, with no significant difference between the two interventions. This is consistent with several other studies that have shown comparable clinical outcomes between telerehabilitation and traditional in-person rehabilitation for knee osteoarthritis and meniscal injuries (11, 12). Tedeschi et al. found that both modes of rehabilitation resulted in similar improvements in function and pain relief for knee osteoarthritis patients, reinforcing the non-inferiority of telerehabilitation in managing musculoskeletal conditions like meniscal tears (13). While many randomized trials showed equivalent outcomes, some narrative reviews and clinical commentaries suggest that face-to-face rehabilitation remains the “gold standard” for patients with very complex needs or when manual techniques are paramount. Cottrell et al., in a systematic review, pointed out that direct physical contact may still be advantageous for complex manual therapies, although the study concluded that there was overall equivalence between telerehabilitation and standard practice (14). Holland also emphasized that hands-on assessment and treatment provided in face-to-face sessions remain critical for some patients (15). Although face-to-face rehabilitation is often considered the gold standard for complex conditions or those requiring extensive manual therapy, our study aimed to define indications for telerehabilitation where protocols are already highly standardized and demand for in-person contact is reduced. Findings from this trial suggest that, with careful selection, middle-aged patients with degenerative meniscal tears in China can achieve satisfactory outcomes through telerehabilitation.

In terms of strength improvement, although the observed differences in peak torque extension at 6 and 12 weeks were not statistically significant and resolved by 24 weeks, the temporary trend favouring the face-to-face group warrants consideration. One plausible explanation is that face-to-face therapy provided immediate tactile feedback, real-time correction of exercise techniques, and possibly greater initial exercise intensity under therapist supervision. In contrast, the telerehabilitation participants relied primarily on visual cues and self-correction, which may have initially slowed strength gains until they became proficient at executing exercises independently.

Clinically, this suggests telerehabilitation programmes might benefit from incorporating more frequent, targeted feedback during the early phases of rehabilitation to ensure exercises are performed optimally, thereby matching the quicker gains seen in traditional settings. Likewise, the cost-effectiveness analysis in this study, which revealed substantial cost savings for the TELE group, aligns with the results of similar studies showing that telerehabilitation reduces healthcare costs by lowering travel expenses, in-person therapy costs, and other related expenses. A systematic review by Guida et al. (16) also concluded that telerehabilitation provides a more cost-effective alternative to traditional rehabilitation methods, supporting our finding of a lower total cost for the TELE group compared with PT.

Our study also demonstrated that the adherence rates were high and comparable across the 2 groups, with both groups showing excellent engagement in their rehabilitation programmes. In total, 12 participants discontinued the study (6 in each group). Five of them dropped out of the study for unrelated health problems, and only 7 participants dropped out of this study otherwise (4.9%, 3 in TELE group and 4 in PT group). This finding is supported by other research showing that telerehabilitation can improve patient adherence due to its convenience and flexibility (17–19). For instance, in a study by Vasavada et al., patients undergoing telerehabilitation for knee osteoarthritis reported similar or higher adherence rates compared with in-person treatment due to the ability to complete exercises at home and at their own pace (20). Similarly, a study by Amin et al. found that telerehabilitation was associated with high patient satisfaction and engagement, which are key factors in successful rehabilitation outcomes (21).

### Clinical implications of the study

This study demonstrates that telerehabilitation for patients with degenerative meniscal tears is clinically effective, offering comparable functional outcomes and patient satisfaction to face-to-face physical therapy, while also providing substantial cost savings. The results have significant clinical implications, particularly in settings where access to in-person rehabilitation may be limited due to geographic, financial, or healthcare system constraints. In countries like China, where vast geographical regions lack sufficient healthcare infrastructure, the implementation of telerehabilitation could help bridge these gaps, ensuring that patients with musculoskeletal conditions receive consistent and effective care without the burden of frequent clinic visits.

The cost-effectiveness analysis is particularly noteworthy. Given the substantial healthcare costs associated with traditional rehabilitation, telerehabilitation presents an opportunity to optimize healthcare resources. It not only reduces transportation and in-person rehabilitation costs but also allows for more personalized and flexible care, leading to better patient engagement and adherence. With increasing reliance on digital health technologies, telerehabilitation could play a pivotal role in reducing healthcare disparities, particularly in low-resource settings (2, 22).

Moreover, the high patient adherence and satisfaction observed in this study underscore the potential of telerehabilitation to enhance the patient experience. Patients in both the TELE group and PT group showed similar levels of engagement, but the TELE group benefited from greater convenience, reducing the logistical barriers often associated with in-person visits. This highlights the importance of providing accessible, patient-centred care that can be delivered remotely, especially in the era of digital health.

This study holds particular significance for the implementation of telerehabilitation in China, where access to healthcare services, particularly in rural or underserved areas, can be limited. The ability to deliver effective rehabilitation remotely has the potential to alleviate the burden on in-person healthcare services and improve access for patients who may otherwise face geographical, financial, or logistical barriers.

### Strengths and limitations

This study has several strengths. It used a rigorous 2-arm, single-blinded randomized controlled trial (RCT) design, minimizing biases and providing reliable results. A broad range of outcome measures, including KOOS, SF-36, muscle strength, functional performance, and cost-effectiveness, were assessed, giving a well-rounded evaluation of both clinical and economic outcomes. The cost-effectiveness analysis highlighted significant savings with telerehabilitation, which could be particularly beneficial in resource-limited healthcare settings. Furthermore, high patient adherence and satisfaction indicate that telerehabilitation is an acceptable and feasible treatment option for patients.

However, there are some limitations. First, our telerehabilitation approach required patients to be comfortable using a smartphone application, potentially limiting generalizability to populations less familiar or less comfortable with digital technology, such as older adults or those with limited digital literacy. Second, while our follow-up period (24 weeks) provided reasonable insights into rehabilitation effectiveness and adherence, it may not fully capture longer-term adherence patterns or potential re-injury rates, which could emerge beyond this timeframe. Lastly, although our study demonstrates feasibility and effectiveness of telerehabilitation within the Chinese context, integrating such interventions into existing healthcare infrastructure – particularly in rural or underserved regions – will require targeted policy support, improved digital infrastructure, dedicated training for local healthcare workers, and collaborations between tertiary hospitals and community healthcare providers to ensure widespread accessibility and sustainability.

In conclusion, this study demonstrates that telerehabilitation is a clinically effective and cost-efficient alternative to face-to-face physical therapy for patients with degenerative meniscal tears. High patient adherence and satisfaction further support its feasibility as a remote rehabilitation option. These findings suggest that telerehabilitation could be a valuable solution in settings with limited access to in-person care, particularly in countries like China, where healthcare accessibility remains a challenge. Further research is needed to explore its long-term effectiveness.

## Supplementary Material





## Trial details

This study was registered in the Chinese Clinical Trial Registry (https://www.chictr.org.cn/).

## Trial registration number

ChiCTR2400094030.

## References

[1] Ahmed I, Radhakrishnan A, Khatri C, Staniszewska S, Hutchinson C, Parsons N, et al. Meniscal tears are more common than previously identified, however, less than a quarter of people with a tear undergo arthroscopy. Knee Surg Sports Traumatol Arthrosc 2021; 29: 3892–3898. 10.1007/s00167-021-06458-2 33521890 PMC8514344

[2] Prvu Bettger J, Green CL, Holmes DN, Chokshi A, Mather RC, 3rd, Hoch BT, et al. Effects of virtual exercise rehabilitation in-home therapy compared with traditional care after total knee arthroplasty: VERITAS, a randomized controlled trial. J Bone Joint Surg Am 2020; 102: 101–109. 10.2106/jbjs.19.00695 31743238

[3] Franco JB, Maximino LP, Barretti Secchi LL, Antonelli BC, Blasca WQ. What are the barriers to telerehabilitation in the treatment of musculoskeletal diseases? Port J Public Health 2024; 42: 33–42. 10.1159/000534762 39469489 PMC11499665

[4] Safran-Norton CE, Sullivan JK, Irrgang JJ, Kerman HM, Bennell KL, Calabrese G, et al. A consensus-based process identifying physical therapy and exercise treatments for patients with degenerative meniscal tears and knee OA: the TeMPO physical therapy interventions and home exercise program. BMC Musculoskelet Disord 2019; 20: 514. 10.1186/s12891-019-2872-x 31684921 PMC6830005

[5] Butcher NJ, Monsour A, Mew EJ, Chan AW, Moher D, Mayo-Wilson E, et al. Guidelines for reporting outcomes in trial reports: the CONSORT-outcomes 2022 extension. JAMA 2022; 328: 2252–2264. 10.1001/jama.2022.21022 36511921

[6] Roos EM, Lohmander LS. The Knee injury and Osteoarthritis Outcome Score (KOOS): from joint injury to osteoarthritis. Health Qual Life Outcomes 2003; 1: 64. 10.1186/1477-7525-1-64 14613558 PMC280702

[7] Ware JE Jr, Sherbourne CD. The MOS 36-item short-form health survey (SF-36). I. Conceptual framework and item selection. Med Care 1992; 30: 473–483. 1593914

[8] Pogetti LS, Nakagawa TH, Conteçote GP, Camargo PR. Core stability, shoulder peak torque and function in throwing athletes with and without shoulder pain. Phys Ther Sport 2018; 34: 36–42. 10.1016/j.ptsp.2018.08.008 30145541

[9] Lam HS, Lau FW, Chan GK, Sykes K. The validity and reliability of a 6-Metre Timed Walk for the functional assessment of patients with stroke. Physiother Theory Pract 2010; 26: 251–255. 10.3109/09593980903015235 20397859

[10] Kishikawa Y, Miyabara H, Uchinoura M, Yamaguchi Y, Nishimura S, Shibata S, et al. Determinants of quality of life in elderly rehabilitation users at a day care service center. J Phys Ther Sci 2023; 35: 12–17. 10.1589/jpts.35.12 36628134 PMC9822824

[11] Supe HM, Mungikar SS, Katage GA, Garg KA, Wani SK. Effect of pain neuroscience education with conventional physiotherapy via telerehabilitation on pain catastrophizing and function in patients with osteoarthritis knee: a randomized controlled trial. J Midlife Health 2023; 14: 123–129. 10.4103/jmh.jmh_33_23 38029040 PMC10664057

[12] Seron P, Oliveros MJ, Gutierrez-Arias R, Fuentes-Aspe R, Torres-Castro RC, Merino-Osorio C, et al. Effectiveness of telerehabilitation in physical therapy: a rapid overview. Phys Ther 2021; 101: pzab053. 10.1093/ptj/pzab053 33561280 PMC7928601

[13] Tedeschi R, Platano D, Pillastrini P, Berti L, Benedetti MG. Effectiveness of tele-rehabilitation in patients with knee osteoarthritis: a randomized controlled trial. Digit Health 2024; 10: 20552076241286186. 10.1177/20552076241286186 39493627 PMC11528740

[14] Cottrell MA, Galea OA, O’Leary SP, Hill AJ, Russell TG. Real-time telerehabilitation for the treatment of musculoskeletal conditions is effective and comparable to standard practice: a systematic review and meta-analysis. Clin Rehabil 2017; 31: 625–638. 10.1177/0269215516645148 27141087

[15] Holland AE. Telephysiotherapy: time to get online. J Physiother 2017; 63: 193–195. 10.1016/j.jphys.2017.08.001 28939309

[16] Guida S, Vitale JA, Swinnen E, Beckwée D, Bargeri S, Pennestrì F, et al. Effects of prehabilitation with advanced technologies in patients with musculoskeletal diseases waiting for surgery: systematic review and meta-analysis. J Med Internet Res 2024; 26: e52943. 10.2196/52943 39666971 PMC11671784

[17] Pak SS, Janela D, Freitas N, Costa F, Moulder R, Molinos M, et al. Comparing digital to conventional physical therapy for chronic shoulder paIn: randomized controlled trial. J Med Internet Res 2023; 25: e49236. 10.2196/49236 37490337 PMC10474513

[18] Zhang Y, Jin Q, Ji C, Yuan P, Chen L. Innovative Telerehabilitation Enhanced Care Programme (ITECP) in young and middle-aged patients with haemorrhagic stroke to improve exercise adherence: protocol of a multicentre randomised controlled trial. BMJ Open 2023; 13: e072268. 10.1136/bmjopen-2023-072268 PMC10749011 38135318

[19] Smiley A, Finkelstein J. Home automated telemanagement system for individualized exercise programs: design and usability evaluation. JMIR Biomed Eng 2024; 9: e65734. 10.2196/65734 39658220 PMC11724215

[20] Vasavada KD, Shankar DS, Avila A, Mojica ES, Hurley ET, Lehane K, et al. Severe attrition and poor satisfaction in patients undergoing telerehabilitation vs. standard in-person rehabilitation after arthroscopic rotator cuff repairs and anterior cruciate ligament reconstructions. Surgeries 2024; 5: 627–639. 10.3390/surgeries5030050

[21] Amin J, Ahmad B, Amin S, Siddiqui AA, Alam MK. Rehabilitation professional and patient satisfaction with telerehabilitation of musculoskeletal disorders: a systematic review. Biomed Res Int 2022; 2022: 7366063. 10.1155/2022/7366063 35958819 PMC9363217

[22] Losina E, Dervan EE, Paltiel AD, Dong Y, Wright RJ, Spindler KP, et al. Defining the value of future research to identify the preferred treatment of meniscal tear in the presence of knee osteoarthritis. PLoS One 2015; 10: e0130256. 10.1371/journal.pone.0130256 26086246 PMC4472814

