# Supplementary material for: TELEREHABILITATION VERSUS FACE-TO-FACE PHYSICAL THERAPY FOR MIDDLE-AGED PATIENTS WITH DEGENERATIVE MENISCAL TEAR IN CHINA: A NON-INFERIORITY RANDOMIZED CONTROLLED TRIAL
Source: J Rehabil Med. 2025 Aug 21;57:43237. doi: 10.2340/jrm.v57.43237 (PMC12398105; doi:10.2340/jrm.v57.43237)
Supplement: Supplementary file 1 [file JRM-57-43237-s1.pdf]

Supplementary material has been published as submitted. It has not been copyedited, or typeset by Journal of Rehabilitation Medicine

Appendix S1

SUPPLEMENTARY MATERIAL AND METHODS

Statistical analysis

The statistical methodology was designed to align with the nature of the data collected, ensuring comprehensive and meaningful analysis for all outcomes. Descriptive statistics were used to summarize baseline demographic and clinical characteristics. Continuous variables were presented as mean ± standard deviation (SD) or median (interquartile range [IQR]), depending on their distribution, while categorical variables were expressed as frequencies and percentages. Group differences at baseline were assessed using independent samples t-tests for normally distributed continuous data, Mann-Whitney U tests for skewed continuous data, and Chi-square tests or Fisher's Exact tests for categorical variables.

Primary and secondary outcomes were analyzed using linear mixed-effects models to account for repeated measures data collected over time. Fixed effects included group (TELE group vs. PT group), time (baseline, 6 weeks, 12 weeks, and 24 weeks), and their interaction (group × time), while age and sex were included as covariates. Random effects were specified to account for within-subject variability, and least-squares means with 95% confidence intervals (CIs) were calculated for group comparisons at each time point. Non-inferiority for the primary outcome, KOOS, was evaluated by comparing the intergroup difference and its one-sided 95% CI to the predefined non-inferiority margin of 10 points, and the predefined non-inferiority margin was based on previous report[1]. For outcomes with single time-point measurements or summary scores, such as peak torque extension and flexion, analysis of variance (ANOVA) was employed, with post-hoc tests conducted to explore significant differences where necessary.

Functional and performance test results, including the One-Leg Hop Test, 6-Meter Timed Hop Test, and Knee Bends in 30 Seconds, were analyzed using linear mixed-effects models for repeated measures, as described above. Within-group comparisons at each time point were performed using paired t-tests or Wilcoxon signed-rank tests when normality assumptions were violated.

Cost-effectiveness analysis involved calculating the incremental cost-effectiveness ratio (ICER) to compare the economic efficiency of telerehabilitation and face-to-face rehabilitation. Costs were assessed based on healthcare resource utilization, absenteeism, and presenteeism data. Bootstrapping with 1,000 replications was used to estimate 95% CIs for cost and effectiveness differences, and results were represented on a cost-effectiveness plane. Additionally, cost-effectiveness acceptability curves (CEACs) were generated to display the probability of telerehabilitation being cost-effective across various willingness-to-pay thresholds.

Missing data were handled using multiple imputation methods to ensure robust results, with sensitivity analyses comparing findings from the imputed data and complete case analyses. Subgroup analyses explored the influence of potential modifiers such as age, sex, and baseline symptom severity on treatment outcomes, while additional sensitivity analyses assessed the robustness of findings under varying assumptions about missing data and adherence.

Rehabilitation Program for middle-aged patients with degenerative meniscal tear

Periods	Goals	Evaluation	Rehabilitation Content	Homework and Remarks
Initial Phase (Weeks 1–4)	Reduce pain and swelling, restore range of motion (ROM), and initiate muscle activation	Baseline KOOS score.<br>Measure knee ROM and strength (e.g., quadriceps isometric strength).<br>Pain scale assessment (VAS or NPRS).	**Heel Slides:**<br>Lie on your back with legs straight. Slowly slide the affected heel towards your buttocks while keeping the heel on the floor, then return to the starting position.<br>Reps: 10–15 per session, 2 sessions/day.<br>**Quadriceps Sets:**<br>Sit with your legs extended. Tighten the quadriceps by pushing the back of your knee towards the floor, hold for 5 seconds, and relax.<br>Reps: 15 repetitions per session, 2 sessions/day.<br>**Straight Leg Raises (SLR):**<br>Lie on your back, tighten the quadriceps of the affected leg, and lift it 30 cm off the floor.<br>Lower it slowly.<br>Reps: 10 repetitions per session, 2 sessions/day.<br>**Ankle Pumps:**<br>Point and flex your foot to encourage blood circulation and reduce swelling.<br>Reps: 20 repetitions, 3 sessions/day.	Perform assigned exercises twice daily.<br>Record exercise completion and symptoms in the app (telerehabilitation) or logbook (face-to-face).<br>Monitor adherence weekly via app usage or therapist feedback.<br>Modify exercises based on swelling and pain levels.
Intermediate Phase (Weeks 5–8)	Improve strength, dynamic stability,	Reassess KOOS and functional test results (e.g., 6-meter timed hop test).	**Mini Squats:**	Complete 30–40 minutes of exercises daily.

	and functional movement.	Measure quadriceps and hamstring strength using dynamometry.	Stand with feet shoulder-width apart, squat down to 45° of knee flexion, and return to standing.<br>Reps: 12–15 repetitions, 2 sessions/day.<br>**Step-Ups:**<br>Step onto a low platform with the affected leg, then back down.<br>Reps: 10–12 repetitions, 2 sessions/day.<br>**Single-Leg Balance:**<br>Stand on the affected leg for 30 seconds.<br>Progress by adding arm movements or unstable surfaces.<br>Duration: 3 repetitions of 30 seconds, 2 sessions/day.<br>**Low-Impact Cardio:**<br>Perform stationary cycling or brisk walking.<br>Duration: 15–20 minutes, 5 days/week.	Engage in light recreational activities such as brisk walking.<br>Focus on proper form and gradually increase resistance.<br>Weekly online consultations or in-clinic feedback to ensure progression.
Advanced Phase (Weeks 9–12)	Enhance functional strength, endurance, and sport-specific skills.	Functional performance tests (e.g., one-leg hop test, knee bends in 30 seconds).<br>Repeat KOOS and SF-36 assessments.	**Plyometric Jumps:**<br>Perform box jumps or lateral hops on both legs, progressing to single-leg hops.<br>Reps: 8–10 repetitions, 3 sets, 3 sessions/week.<br>**Lunges:**<br>Perform forward or walking lunges, keeping knees aligned over toes.<br>Reps: 12–15 repetitions, 3 sets, 3 sessions/week.	Practice sport-specific movements or advanced balance drills.<br>Log physical activity and recovery status.<br>Encourage self-monitoring for fatigue or overuse symptoms.<br>Modify programs to match activity-specific goals.

			**Balance on Wobble Board:**<br>Maintain single-leg balance on a wobble board for 20–30 seconds.<br>Reps: 3 repetitions, 2 sessions/day.<br>**Running Drills:**<br>Start with jogging on flat ground, progressing to short sprints or agility drills.<br>Duration: 15–20 minutes, 3 days/week.	
Maintenance Phase (Weeks 13–24, Instruction only)	Sustain functional gains and prevent recurrence		**Functional Strength Training:**<br>Incorporate weighted squats, lunges, and step-ups.<br>Reps: 12–15 repetitions, 2–3 sets, 3 sessions/week.<br>**Endurance Activities:**<br>Engage in cycling, swimming, or jogging.<br>Duration: 30 minutes, 3–5 days/week.<br>**Advanced Plyometrics:**<br>Include multi-directional hops and drop landings.<br>Reps: 10–12 repetitions, 2–3 sets, 2 sessions/week.	Integrate strength and cardio exercises (e.g., jogging, cycling) into daily routines.<br>Participate in recreational sports with modified intensity if applicable.<br>Encourage long-term adherence through follow-up consultations.<br>Provide educational material on knee health.

SUPPLEMENTARY RESULTS

Cost-effectiveness analysis

Cost-effectiveness analysis revealed that the total average cost per patient over 12 weeks was significantly lower in the TELE group compared to the PT group (56,662.34 ± 10,908.35 CNY vs. 68,616.95 ± 15,072.76 CNY, $p < 0.001$). The primary contributors to cost savings in the TELE group were lower rehabilitation costs (6,980.00 CNY in TELE vs. 10,780.18 ± 1,475.13 CNY in PT, $p < 0.001$), primary care costs (124.24 ± 46.61 CNY in TELE vs. 1,918.18 ± 262.48 CNY in PT, $p < 0.001$), and transportation costs (473.11 ± 188.32 CNY in TELE vs. 1,543.64 ± 282.29 CNY in PT, $p < 0.001$). Medication costs were also slightly lower in the TELE group (7,207.50 ± 1,514.13 CNY vs. 7,883.35 ± 1,348.26 CNY, $p = 0.008$). The costs related to hospital stays and nutrition were comparable between the groups, while lost wages for patients and families did not differ significantly (Table IV).

The incremental cost-effectiveness ratio (ICER) analysis demonstrated that the TELE group was more cost-effective than the PT group. The TELE group had an incremental cost saving of -11,954.61 CNY and a slight but non-significant improvement in KOOS scores (incremental score: 0.483; 95% CI: -0.931 to 1.897). The ICER for KOOS was calculated as -24,750.75 CNY per unit improvement, indicating cost savings with comparable clinical effectiveness. For pain and symptoms subdomains, the TELE group had incremental ICERs of -10,789.36 CNY and -6,902.20 CNY per unit improvement, respectively. Although the TELE group exhibited cost savings across most domains, activities of daily living and function in sport and recreation showed marginally higher ICERs of 16,932.88 CNY and 10,523.42 CNY per unit improvement, respectively (Table V).

Patients' adherence

Patient adherence to treatment was similar between the TELE and PT groups, with no significant differences in key adherence measures. The mean number of sessions performed per week was comparable between the groups (3.3 ± 1.1 sessions for the TELE group vs. 3.4 ± 1.2 sessions for the PT group, $p = 0.425$). Regarding patient agreement with specific treatment aspects, both groups reported similar levels of agreement. The extent to which patients agreed to accept the allocated exercise plan was 8.2 ± 1.2 in the TELE group and 8.4 ± 1.1 in the PT group ($p = 0.620$). Adherence to the recommended exercise program was also similar, with scores of 8.3 ± 1.5 in the TELE group and 8.5 ± 1.6 in the PT group ($p = 0.151$) (Table SI).

Both groups expressed high levels of satisfaction with the interventions. Patients in the TELE group reported a mean satisfaction score of 9.4 ± 0.8, while those in the PT group reported a score of 9.5 ± 0.8 ($p = 0.181$). Additionally, perceptions of pain relief and functional improvement were similarly high in both groups, with scores of 8.5 ± 1.5 and 8.7 ± 1.3, respectively, for pain relief in the TELE group, compared to 8.7 ± 1.3 and 8.9 ± 1.2 in the PT group ($p = 0.916$ and $p = 0.530$, respectively) (Table SI).

Adverse events

Adverse events were reported in both the TELE and PT groups, with a slightly higher incidence in the PT group. A total of 15.3% of patients in the TELE group (11 patients) experienced adverse events, compared to 19.4% in the PT group (14 patients). Among these, 4 patients in the TELE group and 5 patients in the PT group experienced events unrelated

to the study therapy. However, a greater proportion of patients in both groups had events related to the study therapy, with 10 events in the TELE group and 13 in the PT group (Table S2).

The most common adverse events related to the intervention were pain, bruising, and swelling. In the TELE group, 5 patients (3%) experienced pain, 2 patients (2%) reported bruising, and 2 patients (2%) experienced swelling. In the PT group, 7 patients (4%) had pain, 4 patients (4%) experienced bruising, and 3 patients (3%) experienced swelling. Additionally, the TELE group had 1 case of nausea and dizziness, while the PT group had 1 case of anxiety about knee recovery. Other adverse events included pain on the contralateral side and a fall with minor symptoms, with 1 patient in the TELE group reporting pain of the contralateral side, compared to 2 in the PT group (Table S2).

Serious adverse events were relatively rare, with 3 patients (4.2%) in the TELE group and 2 patients (2.8%) in the PT group experiencing such events. Of the serious events, 1 in the TELE group was related to a hip fracture due to a fall, while another patient suffered a severe muscle sprain and severe cartilage degeneration, both related to the study therapy. In the PT group, 1 patient experienced a waist fracture due to a fall, and 1 patient experienced severe low back pain, both unrelated to the study therapy (Table S2).

References

1. Liu JN, Gowd AK, Redondo ML, Christian DR, Cabarcas BC, Yanke AB, et al. Establishing Clinically Significant Outcomes After Meniscal Allograft Transplantation. Orthop J Sports Med 2019;7 (1):2325967118818462.
