# Supplementary material for: TELEREHABILITATION VERSUS FACE-TO-FACE PHYSICAL THERAPY FOR MIDDLE-AGED PATIENTS WITH DEGENERATIVE MENISCAL TEAR IN CHINA: A NON-INFERIORITY RANDOMIZED CONTROLLED TRIAL
Source: J Rehabil Med. 2025 Aug 21;57:43237. doi: 10.2340/jrm.v57.43237 (PMC12398105; doi:10.2340/jrm.v57.43237)
Supplement: Supplementary file 2 [file JRM-57-43237-s2.pdf]

Supplementary material has been published as submitted. It has not been copyedited, or typeset by Journal of Rehabilitation Medicine

Table SI. Patients' adherence to treatment (in intention-to-treat population)

**Outcome measure**	**TELE group (N=72)**	**PT group (N=72)**	**P value**
Number of sessions performed per week, mean ± SD	3.3 ± 1.1	3.4 ± 1.2	0.425
Agreement with the following questions (0 to 10)*, mean (SD)			
To what extent did you agree to accept the allocated exercise plan?	8.2 ± 1.2	8.4 ± 1.1	0.620
To what extent did you do the exercise program as recommended?	8.3 ± 1.5	8.5 ± 1.6	0.151
To what extent do you agree that the intervention relieved your pain?	8.5 ± 1.5	8.7 ± 1.3	0.916
To what extent do you agree that the intervention improved your function?	8.7 ± 1.3	8.9 ± 1.2	0.530
To what extent were you satisfied with the exercise protocol?	9.4 ± 0.8	9.5 ± 0.8	0.181

N/A, not applicable.

* 0 = strongly disagree, 10 = strongly agree.

Table SII. Adverse Events and Serious Adverse Events (in intention-to-treat population)

**Adverse events**	**TELE group (N=72)**	**PT group (N=72)**
Patients with adverse events (no. [%])	11 (15.3)	14 (19.4)
Events unrelated to study therapy (no.)	4	5
Events related to study therapy (no.)	10	13
Type of event (no.)		
Involved knee		
Pain	5 (3*)	7 (4*)
Bruising	2*	4*
Swelling	2*	3*
Other		
Fall with minor symptoms	0	1
Nausea and dizziness	1	0
Pain of contralateral side	1	2 (1*)
Anxiety about knee recovery	3*	1*
**Serious adverse events****		
Patients with serious adverse events (no. [%])	3	2
Events unrelated to study therapy (no.)	2	2
Events related to study therapy (no.)	1	0
Type of event (no.)		
Hip fracture due to fall	1	0
Waist fracture due to fall	0	1
Severe muscle sprain	1*	0
Severe cartilage degeneration	1	0
Severe low back pain	0	1

*Adverse/Serious adverse events related to study therapy

**Patients with serious adverse events were automatically withdrawn from the study
